# Pro-inflammatory Actions of Heme and Other Hemoglobin-Derived DAMPs

**DOI:** 10.3389/fimmu.2020.01323

**Published:** 2020-06-30

**Authors:** Marcelo T. Bozza, Viktória Jeney

**Affiliations:** ^1^Laboratório de Inflamação e Imunidade, Departamento de Imunologia, Universidade Federal Do Rio de Janeiro, Rio de Janeiro, Brazil; ^2^MTA-DE Lendület Vascular Pathophysiology Research Group, Research Centre for Molecular Medicine, Faculty of Medicine, University of Debrecen, Debrecen, Hungary

**Keywords:** hemoglobin, heme, TLR4 (toll-like receptor 4), NLRP3, DAMP, hemolysis (red blood cells)

## Abstract

Damage associated molecular patterns (DAMPs) are endogenous molecules originate from damaged cells and tissues with the ability to trigger and/or modify innate immune responses. Upon hemolysis hemoglobin (Hb) is released from red blood cells (RBCs) to the circulation and give a rise to the production of different Hb redox states and heme which can act as DAMPs. Heme is the best characterized Hb-derived DAMP that targets different immune and non-immune cells. Heme is a chemoattractant, activates the complement system, modulates host defense mechanisms through the activation of innate immune receptors and the heme oxygenase-1/ferritin system, and induces innate immune memory. The contribution of oxidized Hb forms is much less studied, but some evidence show that these species might play distinct roles in intravascular hemolysis-associated pathologies independently of heme release. This review aims to summarize our current knowledge about the formation and pro-inflammatory actions of heme and other Hb-derived DAMPs.

## Introduction

Red blood cells (RBCs)—the most abundant cell type of the human body—deliver oxygen to the tissues during their lifespan of about 120 days. Under homeostasis, destruction of aged RBCs is usually an unrecognized event that takes place in macrophages of splenic and hepatic sinusoids (1). After RBC phagocytosis macrophages efficiently handle the high hemoglobin (Hb) load, break down heme, and redistribute iron for further use, or store it in a catalytically inactive form in ferritin (FT) (1). Some RBCs lyse intravascularly even under physiological conditions releasing Hb and heme to the circulation. To prevent the deleterious effects of extracellular Hb and heme they are scavenged by the acute phase plasma proteins haptoglobin (Hp) and hemopexin (Hx), respectively, and removed from the circulation rapidly (2). On the other hand, oversaturation of this defense system leads to the accumulation of different redox forms of Hb and heme in the circulation.

Heme is a well-known pro-oxidant that feature relies—at least in part—on the ability of heme iron to catalize the generation of hydroxyl-radicals in the Fenton reaction, due to its capability of acting as both an electron donor and an acceptor (3). Another important source of radical species that could mediate heme-induced toxicity is the conversion of organic hydroperoxides (ROOH) into highly reactive alkoxyl (RO•) and peroxyl (ROO•) radicals (4, 5). These radicals trigger lipid peroxidation forming alkyl radicals that in the presence of O_2_ will generate more peroxyl radicals, thus amplifying free radical reactions (6). Finally, heme can also promote reactive oxygen species (ROS) generation through enzymatic reactions mediated by NADPH oxidases (7–11) and by the mitochondria (11).

Besides being a pro-oxidant, three lines of evidence indicated that heme is an inflammatory molecule with unique properties: (i) sterile intra or extra vascular hemolysis cause inflammation, that is controlled by Hx and heme oxygenase-1 (HO-1) (12); (ii) injection of heme in experimental animals triggers local and systemic inflammation (11–14); (iii) heme activates innate immune cells *in vitro* acting as a chemoatractant, inducing cytokine production, ROS generation, and cell death (7, 8, 11, 15, 16). The observations that heme causes macrophage activation dependently of the innate immune receptors TLR4 and NLRP3 were important to a paradigm shift, defining heme as a prototipical damage-associated molecular pattern (DAMP) (11, 16–18). The requirement of TLR4 or NLRP3 to the pathological consequences of experimental sterile hemolysis suggest that heme-induced activation of these pathways contributes to the pathology (11, 12, 19, 20). Importantly, the tissue damage triggered by the actions of labile heme also critically contributes to the pathogenesis of severe infections such as malaria (21–24) and sepsis (14, 25). Growing evidence shows that the complement system can be activated by heme which mechanism play a role in the pathomechanism of certain hemolytic diseases (20, 26–28). On the other hand heme can activate defense mechanisms to establish tolerance and to foster survival of the host in diverse pathological conditions via the induction of the HO-1/FT system (17, 23, 29, 30). Recent investigation showed that heme can induce innate immune memory as well (31).

Growing evidence suggest that besides labile heme other Hb-related DAMPs e.g., metHb, ferrylHb as well as covalently crosslinked Hb multimers can be considered as alarmins (32–34). These species might play distinct, heme-independent roles in intravascular hemolysis-associated pathologies. The multiple mechanisms by which Hb-derived DAMPs modulate cell activation and inflammation, contributing to pathology, are object of intense research. In this review we aim to give an overview of the most recent development of this dynamically evolving field.

## Hb Inside of the RBCs

Hb, the major oxygen-transport protein consist of 2 different subunits, α and β, that compose a α2β2 tetrahedron. Each of the four subunits contains a heme prosthetic group with a central Fe^2+^ (ferrous) ion. Heme iron is critically involved in O_2_ binding. Each ml of human blood contains ~0.3 g of Hb, most of it is compartmentalized within RBCs.

**Figure 1:**
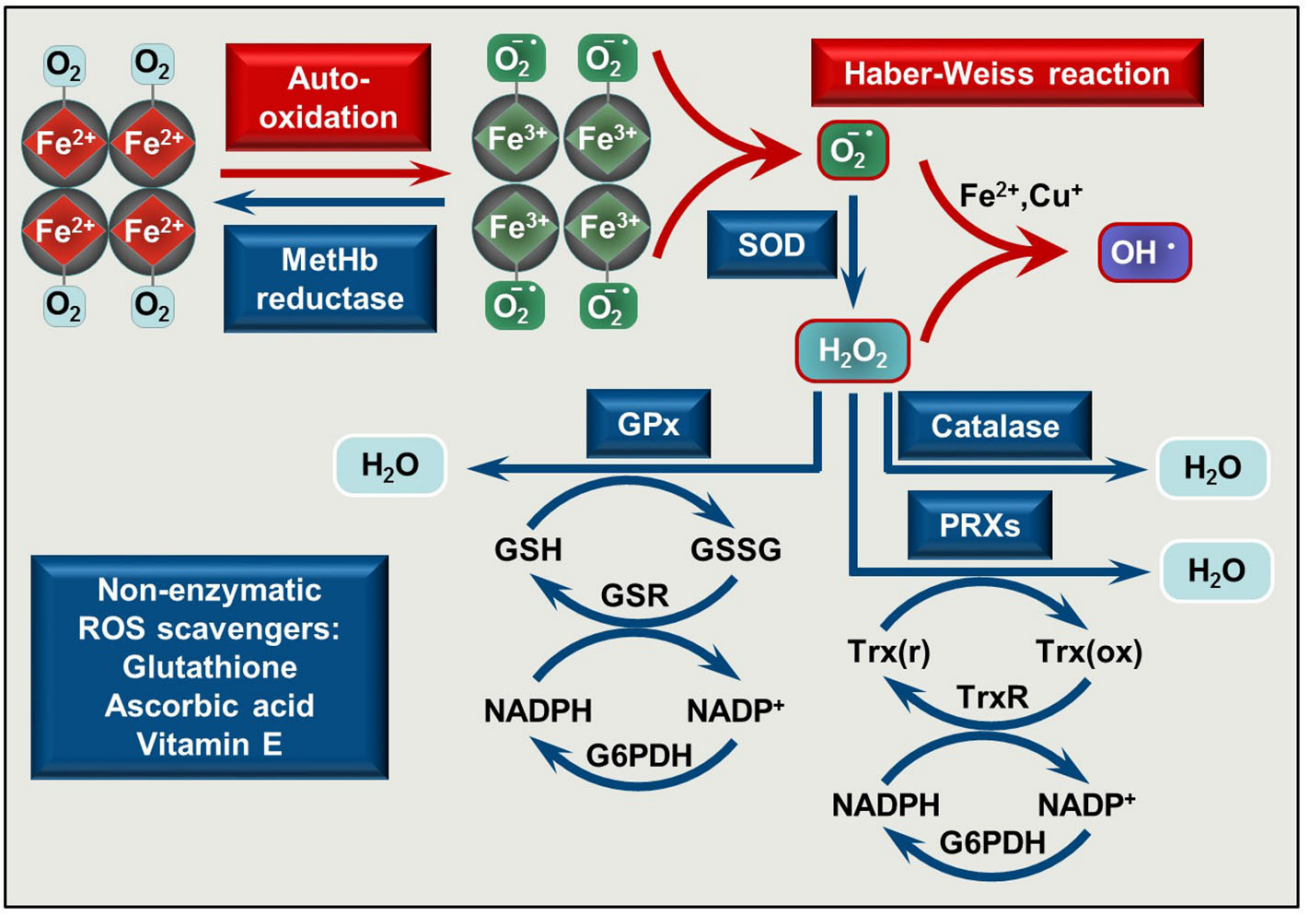
Pro-oxidant and antioxidant mechanisms is RBCs. O_2_ binding to Hb initiates Hb auto-oxidation in which process metHb (Fe^3+^) and superoxide anion (${\text{O}}_{2}^{•-}$) are formed. MetHb is reduced by metHb reductase, while ${\text{O}}_{2}^{•-}$ is converted to H_2_O_2_ by superoxide dismutase (SOD). In the presence of transition metals such as Fe^2+^ or Cu^+^ a reaction between ${\text{O}}_{2}^{•-}$ and H_2_O_2_ occurs yielding hydroxyl radical (OH^•^) (Haber Weiss reaction). Catalase, glutathione peroxidases (GPx), and peroxiredoxins (PRXs) decompose H_2_O_2_. The antioxidant system is completed with non-enzymatic low molecular weight scavengers, such as glutathione, ascorbic acid, and vitamin E. SOD, superoxide dismutase; GPx, glutathione peroxidase; PRXs, peroxiredoxins; GSH, reduced glutathione; GSSG, glutathione disulfide; GSR, glutathione-disulfide reductase; NADP^+^, nicotinamide adenine dinucleotide phosphate; NADPH, reduced NADP; G6PDH, glucose-6-phosphate dehydrogenase; Trx(r), reduced thioredoxin; Trx(ox), oxidized thioredoxin; TrxR, thioredoxin reductase.

Circulating RBCs are continuously exposed to high levels of ROS of both endogenous and exogenous origin [reviewed in (35)]. When Hb binds O_2_, Hb auto-oxidation frequently occurs in which the central heme Fe^2+^ is oxidized into Fe^3+^ (ferric, metHb) with the concomitant reduction of O_2_ into superoxide anion (${\text{O}}_{2}^{•-}$) (Figure 1). This reaction is a major source of endogenous ROS inside the RBCs. Cytochrome-b5 reductase, an NADH-dependent enzyme present in RBCs convert metHb to Hb, therefore metHb content in intact RBC generally stays below 1%.

A highly effective antioxidant defense system protects RBCs from the continuously produced ROS. This system consists of enzymes, such as Cu/Zn superoxide dismutase that converts superoxide anion to hydrogen-peroxide (H_2_O_2_), catalase, glutathione peroxidase, and peroxiredoxins which decompose H_2_O_2_ to H_2_O [reviewed in (35–37)] and non-enzymatic low molecular weight scavengers, such as glutathione, ascorbic acid, and vitamin E (Figure 1). When ROS production exceeds the capability of ROS neutralization, RBC membrane damage occurs which impairs oxygen delivery.

During their lifespan in the circulation RBCs lose about 20% of their initial Hb content via vesiculation (38). This process is considered an efficient mechanism to remove damaged membrane patches, senescent cell antigens and intracellular inclusion bodies (Heinz bodies) from the otherwise healthy RBCs, therefore they can stay longer in the circulation (39). Approximately after 120 days in the circulation RBCs are completely worn out, and they are cleaned from the circulation by hemophagocytic macrophages, mainly in the spleen, via a non-inflammatory process which allows efficient and safe recycling of the RBC components, particularly the heme iron (40–42).

## Hb Outside of the RBCs

Diverse inherited or acquired conditions can trigger uncontrolled destruction of RBCs in the vasculature or in the extravascular space. Upon RBC lysis a large amounts of Hb is released into the circulation, or into the surrounding tissues.

### Elimination of Cell Free Hb and Limitations of the Clearance System

Following RBC lysis extracellular Hb is promptly removed from the circulation. Hp, an acute phase plasma protein is in the first line of defense [reviewed in (43)]. Hp binds extracellular Hb avidly, protects Hb from oxidation (44–48), and facilitates its clearance from the circulation through endocytosis via the CD163 macrophage scavenger receptor (49). Although abundant in the plasma (0.41–1.65 mg/ml), the amount of Hp allows the clearance of ~3 g of Hb, <1% of the Hb amount in the circulation. Therefore, massive hemolysis with more than 1% of RBC lysis, Hp is depleted from the plasma and cell-free Hb is eliminated from the circulation via alternative mechanisms. These include (i) a low-affinity pathway through CD163 by macrophages (50) and (ii) renal excretion which is accompanied by profound oxidative stress and organ damage (51, 52).

### Failure of Hb Clearance: Nitric Oxide Depletion, Hb Oxidation, and Heme Release

**Figure 2:**
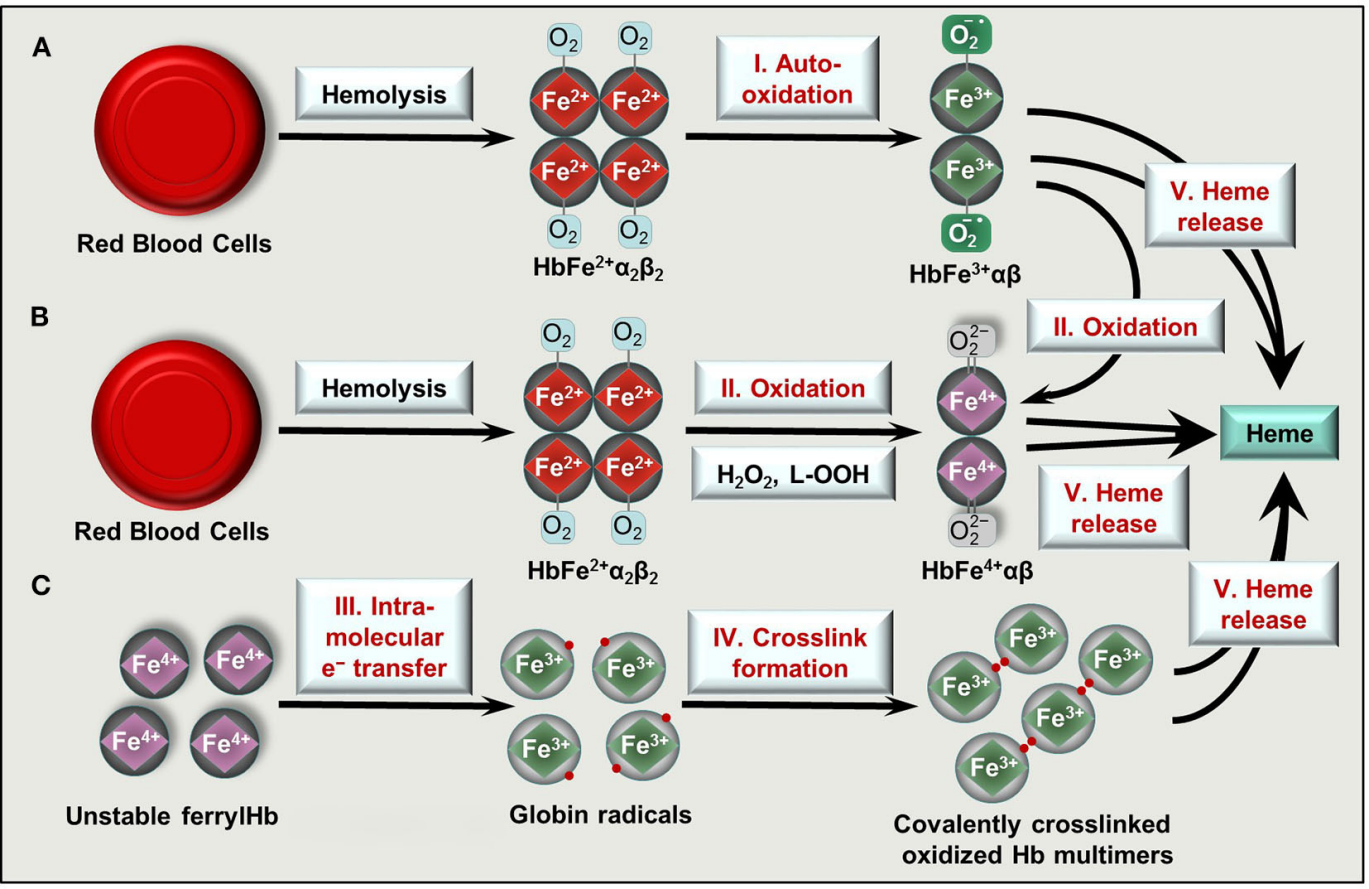
Formation of oxidized Hb forms and labile heme upon hemolysis. Hb tetramers (${\text{HbFe}}^{2+}\alpha_{2}\beta_{2}$) is released from RBCs following intra- or extravascular hemolysis. **(A)** Hb outside of RBCs dimerize and can undergo spontaneous auto-oxidation (reaction I) to metHb (HbFe^3+^αβ). **(B)** Two-electron oxidation (reactions II) of Hb and metHb by H_2_O_2_ or lipid hydroperoxides (L-OOH) lead to the formation of ferrylHb (HbFe^4+^αβ) or ferrylHb radicals, respectively. **(C)** FerrylHb get stabilized via intramolecular electron transfer (reaction III) between iron and the globin chain forming globin radicals. Globin radicals get stabilized via covalent crosslinking (reaction IV) producing covalently crosslinked Hb multimers. Oxidized Hb forms (metHb, ferrylHb, covalently crosslinked Hb) release their heme prosthetic group (reactions V).

Once the capacity of the Hp/CD163 system is overwhelmed, cell free Hb accumulates in the plasma. Hb exhibits a high affinity for nitric oxide (NO), the important endogenously produced gas that plays a major role in the regulation of vascular tone [reviewed in (53, 54)]. Scavenging of NO by Hb triggers vasoconstriction that contributes to clinical complications in diverse forms of hereditary or acquired hemolytic anemias (55). Furthermore, non-compartmentalized Hb cannot benefit from the highly efficient antioxidant defense system present in intact RBCs, and Hb tends to oxidize. One-electron oxidation of Hb occurs when Hb reacts with NO resulting metHb. Also auto-oxidation of oxyHb triggers metHb generation with the concomitant production of superoxide anions (Figure 2A). Two-electron oxidation of Hb occurs when Hb reacts with peroxides, such as H_2_O_2_ or lipid hydroperoxides leading to the formation of ferryl (Fe^4+^ = O ^2−^) Hb (Figure 2B). When metHb reacts with peroxides ferrylHb radical is formed [Hb^•+^(Fe^4+^= O ^2−^)] in which the unpaired electron is located at either the globin chain or at the porphyrin ring (56–59). The ferryl oxidation state of iron is very unstable, therefore these high-valence Hb forms are short-lived intermediates that decay quickly (60).

Ferryl iron can oxidize the neighboring oxidation-prone amino acid residues of the globin chains (i.e., αTyr-24, αTyr-42, αHis-20, βTyr-35, βTyr-130, and βCys-93) with the concomitant reduction of Fe^4+^ into Fe^3+^ (37, 61, 62). This intramolecular electron transfer between the ferryl iron and the amino acids yields metHb globin radicals in which the unpaired electrons are located on the oxidized amino acid residues (37, 61, 62). Reactions between globin radicals or between globin and porphyrin-centered radicals lead to the formation of globin-globin and porphyrin-globin adducts, respectively (Figure 2C). These structurally altered Hb forms are less efficiently removed from the circulation because both high-affinity (Hb-Hp/CD163) and low-affinity (Hb/CD163) endocytosis pathways are compromised (50, 63).

The prosthetic heme group is tightly bound in Hb, while this bound is weakened in oxidized Hb forms. Both metHb and ferrylHb releases heme moiety (Figure 2) which is captured by the acute phase plasma protein Hx (64). Hx-heme complexes are taken up mainly by macrophages and hepatocytes through the scavenger receptor LDL receptor-related protein 1/CD91 (65, 66). Similarly to Hp, Hx is also depleted from the plasma upon massive intravascular hemolysis, leading to the appearance of labile heme, that is, a redox active form of heme which is loosely bound to molecules, other than hemoproteins including albumin, α_1_-microglobulin and lipoproteins such as LDL and HDL.

Recycling of heme iron is a critical component of systemic iron metabolism. Iron is released from the heme molecule via the action of heme oxygenases (HOs), mainly HO-1, the inducible isoenzyme that catabolizes free heme into equimolar amounts of Fe^2+^, carbon monoxide (CO), and biliverdin (67). HO-1 induction and heme degradation products exhibit various cytoprotective mechanisms (29). Heme-mediated HO-1 induction and iron release is associated with the upregulation of ferritin, the major intracellular iron storage protein, assuring that iron is stored in a catalytically inactive still bioavailable form inside the cells (68).

## Activation of the Innate Immune System by Labile Heme and Oxidized Hb Forms

**Figure 3:**
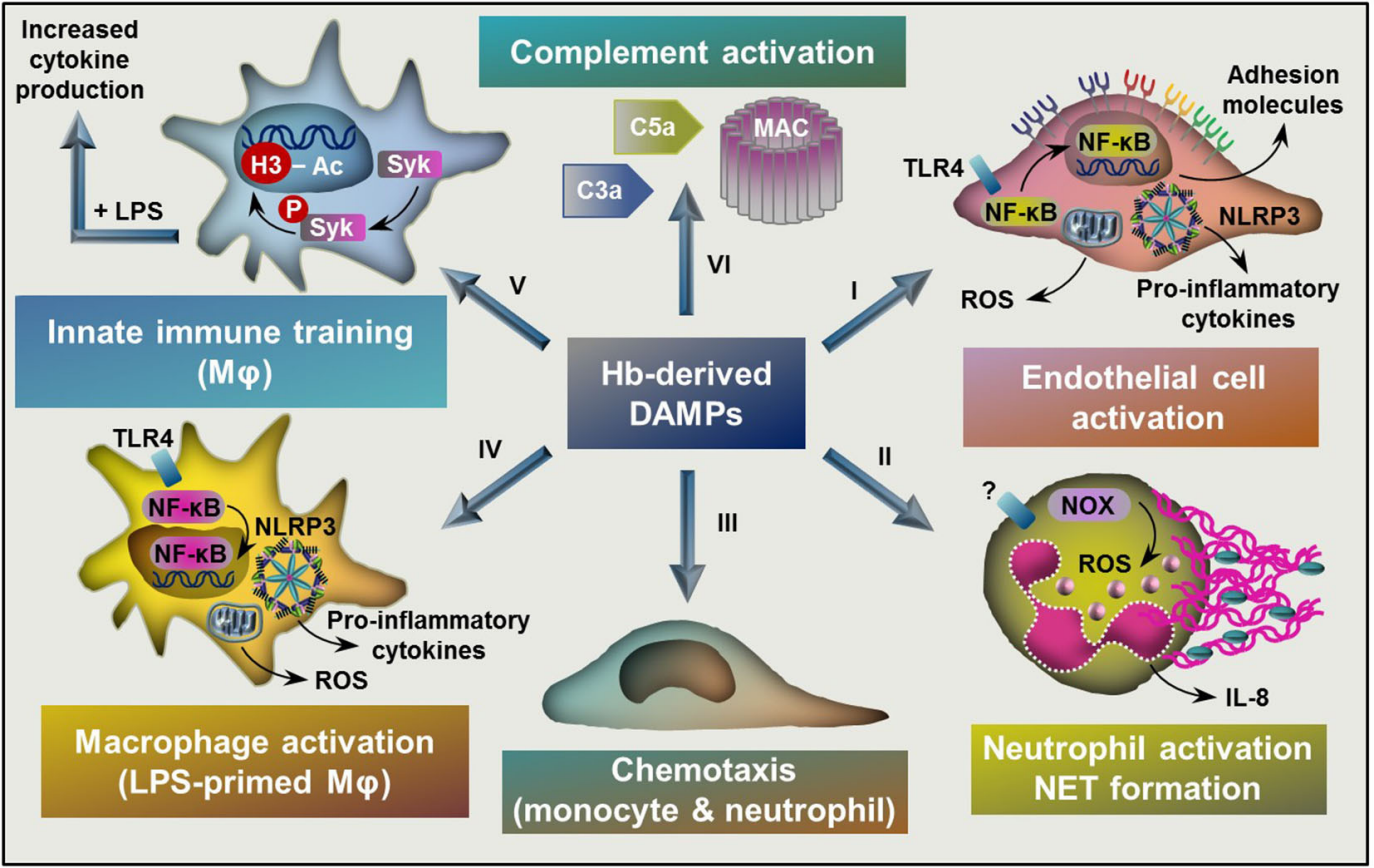
Targets of Hb-derived DAMPs. (I) Labile heme and ferrylHb induces endothelial cell activation characterized by NF-κB activation, elevated ROS production, and increased expression of adhesion molecules and pro-inflammatory cytokines. (II) Heme activates neutrophils characterized by elevated ROS production through the activation of NOX, increased production of IL-8 and NET formation. (III) Heme and ferrylHb induces monocyte and neutrophil chemotaxis. (IV) Labile heme and ferrylHb induces ROS production, NLRP3 activation, and pro-inflammatory cytokine production in LPS-primed macrophages. (V) Heme induces innate immune training through triggering epigenetic changes, such as acetylation of H3 at lysine-27 in monocytes and macrophages in a Syk-dependent manner. (VI) Heme induces complement activation leading to the formation of C3a and C5a activation fragments and the assembly of MAC. NF-κB, nuclear factor kappa B; ROS, reactive oxygen species; NOX, NADPH oxidase; NET, neutrophil extracellular trap; TLR4, toll-like receptor 4, NLRP3, NLR family pyrin domain containing 3; LPS, lipopolysaccharide; Syk, Spleen tyrosine kinase; H3, histone 3, MAC, membrane attack complex.

Hemolytic and hemorrhagic episodes are often accompanied by inflammation even in the absence of pathogens (17, 69). Accumulating evidence suggest that upon hemolysis RBCs release large amounts of DAMPs including RBC microvesicles, heme, ATP, heat shock protein 70, interleukin-33 that induce pro-inflammatory responses in different cells (70, 71). Here we will focus on the contribution of Hb-derived DAMPs to the hemolysis-induced sterile inflammatory responses (Figure 3).

### Endothelial Cells (ECs) as First Line Targets of Hb-Derived DAMPs

A monolayer of ECs cover the entire vasculature and the lymphatic system providing a semi-permeable barrier between blood and tissue, and lymph and tissue, respectively. Under physiological conditions, ECs are involved in many processes including the regulation of metabolic homeostasis, vascular hemodynamics, vascular permeability, coagulation, and cell trafficking [reviewed in (72)]. Besides of these numerous functions, ECs are one of the first cell types to detect pathogen-associated molecular patterns (PAMPs) and DAMPs in the bloodstream, therefore ECs have important immunological functions in the early innate immune system activation as danger signal sensors [reviewed in (72)]. ECs are equipped with a series of pathogen-associated pattern recognition receptors (PRRs) including toll-like receptors (TLRs) and nucleotide-binding oligomerization domain (NOD)-like receptors (NLRs), as well as diverse chemokine receptors [reviewed in (73)]. Growing evidence shows that ECs respond to various Hb-derived DAMPs.

#### Heme-Mediated TLR4-Dependent EC Activation (Adhesion Molecules and Barrier Function)

ECs respond to a variety of inflammatory stimuli e.g., IL-1, tumor necrosis factor α (TNF-α), lipopolysaccharid (LPS) by upregulating the expression of cellular adhesion molecules including intracellular adhesion molecule-1 (ICAM-1), vascular cell adhesion molecule-1 (VCAM-1), and E selectin (74, 75). These adhesion molecules anchor leukocytes to the endothelial surface and facilitate their transmigration into the inflamed tissue. Interestingly heme, similarly to that of IL-1, TNF-α, or LPS upregulates the expressions of adhesion molecules (76) in a TLR4-dependent manner (19, 20).

This heme-mediated TLR4-dependent mechanism has been connected to vaso-occlusive crisis in sickle cell disease (Figure 3) (19). Recent evidence shows that TLR4-dependent upregulation of endothelial P-selectin triggers an unconventional route of complement activation by non-covalent binding of C3 activation fragments on the surface of ECs, which mechanism contributes to liver injury in hemolytic diseases such as sickle cell disease (20).

Besides increased expression of cell surface adhesion molecules, increased endothelial permeability contributes to inflammatory cell extravasation upon hemolysis. Many attempts were made to identify the molecular mechanism of this phenomenon which revealed that ferrylHb and free heme trigger the loss of endothelial integrity (33, 77–80). Heme-induced loss of endothelial barrier function is dependent on the activation of the p38/heat shock protein 27 pathway (79) and associated with TLR4-dependent production of ROS and necroptosis (77).

#### FerrylHb-Mediated TLR4-Independent EC Activation (Adhesion Molecules and Barrier Function)

Besides heme, ferrylHb but not Hb or metHb induces up-regulation of adhesion molecules ICAM-1, VCAM-1, and E-selectin, and increase endothelial cell monolayer permeability in human ECs (Figure 3) (33). Interestingly, ferrylHb-mediated responses are dependent on the activation of nuclear factor kappaB (NF-κB), requires actin polymerization, involves the activation of the c-Jun N-terminal kinase and the p38 mitogen-activated protein kinase signal transduction pathways but not dependent on TLR4 activation (33). The facts that (i) ferrylHb and heme trigger endothelial activation with the use different signaling mechanisms and that (ii) metHb – that can release heme more avidly than ferrylHb – does not induce EC activation suggest that ferrylHb-mediated EC activation cannot be simply considered as a consequence of heme release from ferrylHb. The putative receptors associated with ferrylHb-induced EC activation are currently unknown.

#### Induction of Cytokine Production by Hb-Derived DAMPs in ECs

Growing evidence suggest that endothelial cells are not only sentinels of the activation of the innate immune system but actively participate in cytokine production upon hemolysis. Recently heme and ferrylHb were identified as activators of the NLRP3 inflammasome leading to processing and secretion of active IL-1β in LPS-primed macrophages (11, 34). Previously it has been shown that ECs respond to classical DAMPs such as extracellular ATP and high mobility group box 1 protein (HMGB1) by the activation of NLRP3 inflammasome and the subsequent production of IL-1β (81, 82). Based on these information we investigated whether heme and the different Hb redox forms induce NLRP3 inflammasome activation in ECs (32). We showed that heme but not the different Hb redox forms induced NLRP3 inflammasome activation and IL-1β production ECs (Figure 3). Heme-induced inflammasome activation in ECs requires LPS priming, structural integrity of the heme molecule, and ROS production (32). Recent data suggest that globin-derived peptides formed during Hb oxidation are also capable to induce NLRP3 inflammasome activation and IL-1β production in ECs (83). Besides IL-1β production, Hb-derived DAMPs, namely metHb but not Hb has been implicated in IL-6 and IL-8 production in ECs (84). In the same experimental setting heme did not induce IL-6 and IL-8 production in ECs, so we can assume that metHb-induced EC response was independent of heme release.

### Hb-Derived Molecules as Chemoattractants

Heme is chemoatractant *in vivo*, which notion is supported by the finding that peritoneal injection of heme causes the recruitment of neutrophils and intravenous administration of heme causes leukocyte infiltration in various organs (7, 11, 15, 85). Heme-induced neutrophil recruitment is independent of TLR4 activation (16), but depends on the endogenous production of leukotriene B4 by macrophages (86) and the activation of the NLRP3 inflammasome (11).

Recent studies showed that besides heme, ferrylHb, but not Hb and metHb triggers peritoneal infiltration of monocytes and neutrophils (33, 34). The chemotactic effect of ferrylHb is less likely to be dependent on heme release exclusively, which is supported by two facts; first, ferrylHb is a more powerful inducer of leukocyte infiltration than heme and second, metHb that has the ability to release heme at the same or even higher rate as ferrylHb (87) fails to trigger leukocyte recruitment (34).

### Actions on Neutrophils

Neutrophil granulocytes play a fundamental role in innate and adaptive immunity. Upon infection or inflammation, neutrophils are the first leukocytes migrating from the blood into the affected tissues. Neutrophils are equipped with sensors of PAMPs and DAMPs, they kill and phagocytose pathogens and clear cellular debris (88). In the recent years, it has become evident that neutrophils not only sense PAMPs but can recognize and respond to endogenous DAMPs as well. As it was mentioned before, heme and ferrylHb are potent triggers of neutrophil infiltration (7, 15, 33, 85). Moreover, heme has been shown to activate neutrophils characterized by elevated ROS production and increased expression of the pro-inflammatory cytokine IL-8 (Figure 3) (7, 15). Heme is a potent chemoattractant of neutrophils *in vitro* in a mechanism characteristic of a G protein-coupled receptor activation (7, 15). Heme-induced neutrophil chemotaxis and ROS production are independent of the coordinated iron present in heme, while requires the vinyl groups in the porphyrin ring (15, 89).

Upon activation neutrophils release extracellular traps—meshes composed of chromatin and neutrophil granular proteins—which plays a critical role in immobilization of invading pathogens (90). Recently heme has been identified as a potent inducer of neutrophil extracellular trap (NET) formation in TNF-α-primed neutrophils *in vitro* and *in vivo* (91). Accumulating evidence show that heme-mediated NET formation plays a pathogenic role in vaso-occlusion crises in sickle cell disease, in transfusion-related acute lung injury, in systemic inflammation in paroxysmal nocturnal hemoglobinuria as well as in malaria (91–94). Interestingly, heme-induced NET formation requires the coordinated heme iron, is dependent on NADPH oxidase and ROS formation but occurs independently of TLR4 (95). These results suggest that at least two different signaling pathways are activated by heme on neutrophils. One that triggers chemotaxis and is independent of the heme iron while requires the vinyl groups, and one that triggers the NET release, requires the iron but not the vinyl groups of the porphyrin ring. The putative receptors associated with these activities are currently unknown.

### Activation of Macrophages by Hb-Derived DAMPs

Macrophages are effector cells of the innate immune system, which respond to a variety of PAMPs and DAMPs. Macrophages are present in all vertebrate tissues and have highly heterogeneous phenotypes depending on the environmental cues encountered.

#### TLR4 Activation by Heme in Macrophages

Heme profoundly affect macrophage physiology through multiple pathways (17, 18). The requirement of TLR4 to the induction of TNF production by heme on macrophages was the first demonstration of a receptor-mediated effect of heme (16). The coordinated iron and the vinyl groups are essential for heme to induce TLR4-dependent TNF production. The effect of heme on macrophages through TLR4 is exquisitely different from the effect of LPS, the canonical agonist of TLR4. While LPS triggers the activation of the Myeloid differentiation primary response 88 (MyD88) and the TIR-domain-containing adapter-inducing interferon-β (TRIF) pathways (96) on macrophages and DCs, heme activates only the MYD-88 pathway and is unable to induce the expression of type I interferon or co-stimulatory molecules (16).

#### Heme-Induced Macrophage Necroptosis

High amounts of free heme due to hemolysis is involved with the loss of macrophages, specially in the absence of HO-1 (97). Heme induces macrophage necroptotic cell death in a mechanism that requires TNF, ROS, and the kinases RIPK1 and RIPK3 (98). Although required to heme-induced TNF production, TLR4 is not essential for the necroptosis induced by heme in the presence of exogenous TNF. An important study demonstrated that heme triggers tissue macrophage differentiation by inducing the transcriptional factor Spic in monocytes through a mechanism dependent on the degradation of the transcriptional repressor Bach1 (99). During pathological hemolysis, the axis formed by heme, Bach1, and Spic is critically involved in the homeostatic response to the macrophage loss. Another compensatory response to hemolysis and heme is the formation of aggresome-like induced structures (ALIS) on macrophages, p62/SQTM1 aggregates containing ubiquitinated proteins (100, 101). The heme-induced ALIS formation on macrophages requires mitochondrial ROS, NRF2 and HO-1, while is independent of TLR4. Moreover, iron from heme is necessary, while both Fe^2+^ and Fe^3+^ are sufficient to trigger ALIS formation (100). The physiopathological role of heme-iduced ALIS formation is currently unknown.

#### NLRP3 Activation by Heme in Macrophages

Heme has a synergistic effect with microbial molecules on macrophages, increasing the production of inflammatory cytokines in a mechanism dependent of ROS and spleen tyrosine kinase (Syk) (13). Moreover, heme induces NLRP3 inflammasome activation leading to processing and secretion of active IL-1β in LPS-primed macrophages (11). Heme-mediated NLRP3 inflammasome activation is found to be dependent on the coordinated heme iron, and also involves activation of Syk, elevated ROS production by NOX2 and the mitochondria and K(+) efflux, contributing to intravascular hemolysis-induced lethality (11). An interesting study demonstrated that heme reduces the host resistance to bacterial infection (102). Treatment of macrophages with heme, but not with Hb, free iron, or the heme analogs protoporphyrin IX and tin-protoporphyrin IX, causes a dose-dependent inhibition of *E. coli* phagocytosis by macrophages (102). This inhibitory effect of heme on macrophage phagocytosis and chemotaxis occurs through the activation of the GTP-binding Rho family protein Cdc42 by DOCK8, a guanine nucleotide exchange factor, disrupting actin cytoskeletal dynamics (102). Together, these results indicate possible signaling pathways for therapeutic intervention during hemolytic infectious conditions.

#### NLRP3 Activation by ferrylHb in Macrophages

Besides heme, the involvement of different Hb redox forms was investigated in hemolysis-associated NLRP3 inflammasome activation in macrophages. That study revealed that ferrylHb but not Hb or metHb induce active IL-1β production in LPS-primed macrophages in an NLRP3-dependent manner (34). Based on the fact that metHb cannot induce IL-1β production in LPS-primed macrophages it is unlikely that heme release plays a critical role in the ferrylHb-triggered response. FerrylHb-induced NLRP3 activation is associated with elevated ROS production but the detailed molecular mechanism needs to be further explored (34).

#### Hemorrhage-Associated Macrophage Subsets

Besides the two extreme canonical macrophage phenotypes, the pro-inflammatory M1 and the anti-inflammatory M2, many other specific and distinct macrophage subsets exist (103). Both extracellular Hb and heme are implicated in macrophage polarization triggering the formation of hemorrhage-associated M(Hb) and M(heme) subsets, respectively (103–106). These hemorrhage-associated macrophage subsets were first identified in advanced human atherosclerotic lesions with intraplaque hemorrhage (103–106). M(Hb) macrophages represent a subpopulation of CD68+ macrophages and their characteristic markers are the macrophage mannose receptor 1 and the CD163 receptor through which macrophages recognize and endocytose Hp-Hb complexes (103–105). Additionally, due to their role in Hb clearance, M(Hb) macrophages exhibit increased HO-1 and ferroportin expressions thereby facilitating heme catabolism and cellular efflux of excess iron (105). Reduced labile iron content in M(Hb) macrophages is associated with less ROS production which is linked to increased activity of the transcription factor liver X receptor-α and the induction of cholesterol efflux (105). Because of this, M(Hb) macrophages are protected from lipid accumulation and produce anti-inflammatory factors, such as IL-10 (105). M(heme) macrophage polarization is driven by extracellular heme, and similarly to that of M(Hb) this subset is protected from oxidative stress and lipid accumulation (107).

### Activation of Microglia by Hb-Derived DAMPs

Microglia are the primary innate immune effector cells of the central nervous system (CNS) with a similar function to macrophages. Intracerebral and subarachnoid hemorrhages (ICH and SAH, respectively) are associated with activation of microglia and growing evidence suggest that inflammation is the key contributor of secondary brain injury induced by ICH or SAH (108, 109). Microglia have an important function in hematoma resolution by phagocytosing RBCs which process is mediated by the class B scavenger receptor CD36 (110, 111). CD36 expression is regulated by peroxisome proliferator-activated receptor γ (PPARγ), and activation of PPARγ has been shown to promote hematoma resolution and decrease neuronal damage following ICH (111). Incomplete removal of RBCs leads to hemolysis and the production of oxidized Hb forms and free heme (112, 113). Microglia plays a critical role in removing these toxic Hb derivatives from the central nervous system through CD163-Hp-Hb and CD91-Hx-heme scavenging mechanisms and heme degradation by HO enzymes (114–117). These mechanisms can attenuate bleeding-associated neuronal damage, though we have to note that upon significant intrathecal hemolysis the Hb-heme elimination system is largely overwhelmed leading to the accumulation of oxidized Hb forms and free heme in the CNS (112–114).

These Hb-derived DAMPs trigger neuroinflammation following ICH and SAH. In line of this notion it has been shown that heme induces TLR4-mediated inflammatory injury via the activation of MyD88/TRIF pathways in microglia following ICH (118). Importantly, mice deficient of *Tlr4* or anti-TLR4 treatment reduce heme-induced neurologic deficit, brain edema, and inflammation. Not only heme, but also metHb has been identified as a TLR4 agonist that triggers the secretion of TNF-α by the microglia (119). A number of studies support the notion that TLR4 contributes to the brain injury due to ICH (120–123). Additionally, heme induces the release of IL-1α but not IL-1β in primary mixed glia (124). Targeting these inflammatory pathways with anti-TLR4 antibody or with IL-1 receptor antagonist attenuate intrathecal hemorrhage-associated inflammatory injury (118, 124). A recent study showed that besides heme and metHb, large amounts of covalently crosslinked Hb multimers (dimers and tetramers) accumulate in the cerebrospinal fluid of preterm infants following IVH (112). Further work needed to address whether these Hb multimers are implicated in the inflammatory microglia activation following IVH.

### Induction of Innate Immune Memory by Heme

Trained immunity or innate immune memory is the ability of the innate immune system to adapt its function after previous encounters with pathogens or their products (125). This mechanism not only provides protection against reinfection but also contributes cross-protection between infections with different pathogens (125, 126). The major cell types in which trained immunity occurs are myeloid cells, natural killer cells, and innate lymphoid cells. Trained immunity is activated by PAMPs such as LPS or β-glucan via PRR signaling, resulting changes in transcription programs through epigenetic regulation that can persist for up to several weeks (125, 126). Epigenetic reprogramming – driven by histone acetylation, for example at lysine-9 (H3K9ac) and lysine-27 (H3K27ac) of H3 histones that almost exclusively determines transcriptional capability – is a critical determinant of trained immunity (127).

Recently it was reported that heme is a potent inducer of trained immunity in monocytes and macrophages both *in vitro* and *in vivo* (31). Heme pretreatment increased pro-inflammatory cytokine (TNF-α, IL-6, IL-8) release from macrophages upon secondary challenge by LPS (Figure 3) (31). Such effect of heme was independent on the pro-oxidant nature of heme, which notion is supported by the fact that (i) trained immunity is induced by protoporphyrin IX, lacking iron and (ii) it is not prevented by the glutathione precursor N-acetyl cysteine (31). Heme pretreatment triggered epigenetic changes, such as acetylation of H3k27. Comparing heme and β-glucan-induced training in monocytes revealed overlapping as well as distinct epigenetic and transcriptional responses between the two triggers (31). Common pathways, regulated by both heme and β-glucan included lysosome maturation and metabolism. Genes only induced by heme are mainly involved in inflammatory pathways, and as expected heme/iron related metabolism (31). Another remarkable difference between heme- and β-glucan-induced training is that heme-mediated training relies on the activation of Syk and c-Jun n-terminal kinase, but independent on the activation of the Mammalian Target of Rapamycin which is largely involved in β-glucan training (31). This finding reinforces the critical role of Syk signaling on heme-induced macrophage activation (11, 13). At present, the mechanism by which heme triggers Syk phosphorylation is unknown (17). Interestingly, heme seems to be a Janus-faced training molecule *in vivo* resulting that the outcome of heme pretreatment largely depends on the experimental conditions (31).

### Complement Activation and the Thromboinflammatory Loop

Originally the complement system has been considered as a simple mechanism to induce bacterial lysis. Recently it became evident that complement has diverse functions in both physiologic and pathologic conditions (128). The complement system senses PAMPs and DAMPs and translate the danger information into an adequate cellular innate or adaptive immune response (129). The complement system is a cascade of more than 40 proteins, which can be initiated by different ways. There are three known distinct ways for complement activation: the classical, the lectin-mediated, and the alternative pathway (AP) (130). Activation of each of the three pathways leads to a common terminal pathway in which the inactive C3 protein is cleaved into the functional fragments C3a and C3b, and the membrane attack complex (MAC) is formed (130). These products of complement activation mediate a diverse inflammatory response that includes opsonization and phagocytosis, bacterial killing, immune cell recruitment, endothelial and epithelial cell activation, platelet activation and interaction with the adaptive immune system (128).

It has long been known that hemolytic disorders, such as sickle-cell disease, beta-thalassemia major, thrombotic thrombocytopenic purpura, and paroxysmal nocturnal hemoglobinuria are associated with complement over-activation (131–136). Recent evidence suggest that heme has a direct role in hemolysis-associated complement activation (Figure 3). In line of this notion heme has been shown to activate the complement AP and trigger the deposition of C3 activation fragments on the surface of RBCs (28). A detailed work showed that C3a, C5a, and sC5b9 activation fragments are formed during heme-mediated activation of the complement AP in normal human serum (Figure 3) (26). Additionally, heme-exposed ECs also activate the AP resulting in cell-bound C3 and MAC, which mechanism contributes to endothelial damage and thrombosis in atypical hemolytic uremic syndrome (26). Drug-induced intravascular hemolysis or injection of heme trigger C3 deposition in the kidneys and subsequent renal damage which can be attenuated by the heme scavenger Hx. Also, deficiency of C3 attenuates hemolysis-induced kidney injury in mice, suggesting that heme-mediated complement activation and C3 deposition play a fundamental role in renal damage upon intravascular hemolysis (27).

Clinical and epidemiological studies revealed that RBC abnormalities such as abnormal hematocrit, sickle cell disease, thalassemia, hemolytic anemias, and malaria are associated with increased incidence of both arterial and venous thrombosis (137). Recently it has been shown that heme activates the tissue factor (TF)-dependent extrinsic coagulation pathway both *in vitro* and *in vivo* which can be attenuated by an anti-TF antibody (138, 139). Importantly, inhibition of TF-induced coagulation activation reduces microvascular stasis and lung vaso-occlusion in sickle mice (140). By activating both inflammatory and hemostatic pathways extracellular heme can trigger a thromboinflammatory vicious cycle that can contribute to the pathogenesis of hemolytic diseases. Recently the heme-induced thrombogenecity was studied in an *ex vivo* human whole blood model. Heme-induced thromboinflammation was attenuated by the inhibition of the complement component C5 and the TLR coreceptor CD14 (141). Inhibition of the thromboinflammatory loop can be a meaningful therapeutic target in hemolytic diseases (141).

### Modulation of Host Defense Mechanism by Labile Heme

High concentrations of labile heme are observed in infectious conditions, such as malaria and sepsis, both in humans and experimental animals (14, 17, 21, 29). As in sterile hemolytic conditions, the axis Hp/Hx and HO-1/FT heavy chain (FTH) also provides critical host protection against free heme on infectious disease with increased hemolysis (14, 21–25, 29). Two complementary mechanisms comprise the host response to infection. Resistance, a primarily attribute of the immune system, is associated with the reduction or elimination of infectious agents, while tolerance is the capacity of limiting the pathological consequences of an infection (142, 143). Heme can affect the host responses in multiple ways, modulating both the disease tolerance and the resistance to infection. In a series of important studies, it has been demonstrated that heme contributes to the pathogenesis of malaria by increasing tissue damage, while HO-1 and FTH contributes to disease tolerance irrespective of changes on pathogen loads (21–24). A recent study indicates that sickle cell trait with low grade hemolysis is beneficial in malaria infection due to an increased in disease tolerance associated with higher HO-1 expression (23). CO, generated by the catabolism of heme by HO-1, binds to heme inhibiting its release from the Hb during malaria, thus preventing pathology (21). Moreover, the antioxidant effects of HO-1 inhibits the hepatocyte apoptosis induced by the synergistic effects of heme and TNF, preventing hepatic failure, and death in a mouse model of malaria (144).

In a mouse model of endotoxemia heme enhances the plasma concentrations of TNF and IL-6, drastically increasing the lethality induced by LPS (13). This increased lethality in mice is observed even when the challenge with LPS occurs after 6 days of treatment with heme and correlates with increased numbers of tissue macrophages (31). Heme reduces blood glucose levels dependently of TLR4, contributing to the severity of sepsis, while FTH reverts this effect contributing to glucose and tissue homeostasis (25). In mouse models of severe bacterial infection, heme increases multi organ failure, and lethality irrespective to a change on pathogen load (14). An interesting study has shown that heme reduces resistance to Gram-negative infection in mice predisposing to pathogen dissemination through the suppression of phagocytic function and independently of bacterial growth due to nutritional advantage (102). These results suggest that upon acute bacterial infection heme can be deleterious due to an increase on tissue damage and bacterial loads.

Heme can also modify immunoglobulin-mediated immune responses. This activity relies on the ability of heme to bind to immunoglobulins of different isotypes (IgG, IgA and IgM) leading to the formation of heme-immunoglobulin complexes that exhibit increased reactivities toward various self and bacterial antigens (145). Besides that, it has been shown that heme-IgG complex can interact with previously unrecognized bacterial antigens and intact bacteria through binding to an enlarged panel of structurally unrelated epitopes (146). Heme-induced expansion of the antibody repertoire may represent an inducible innate-type host defense mechanism against infections (146).

## Conclusions

Upon hemolysis a large amount of Hb is released from RBCs that is oxidized in the extracellular milieu. Cell free Hb, its oxidation products and heme that is released from oxidized Hb forms are potential DAMPs. Among these numerous Hb oxidation products heme is the most widely studied molecule, and its contribution as a DAMP in hemolysis-associated pathologies has been confirmed. Because of structural alterations oxidized Hb forms (metHb and ferrylHb) bind heme less avidly than Hb, therefore pro-inflammatory actions of oxidized Hb forms was thought to be attributed to their ability to release the heme prosthetic group. This idea is challenged by recent studies suggesting that oxidized Hb forms, in particular ferrylHb exhibit pro-inflammatory actions independently of heme release. A lot of work needs to be done to further explore the colorful picture of Hb-derived DAMPs, their targeted cells and the mechanisms of their actions. Comprehensive understanding of hemolysis/hemorrhage-associated inflammation could contribute to the development of novel therapeutics intended to interrupt these pathological events.

## Author Contributions

MB and VJ planned and wrote the manuscript. VJ draw the figures. All authors contributed to the article and approved the submitted version.

## Conflict of Interest

The authors declare that the research was conducted in the absence of any commercial or financial relationships that could be construed as a potential conflict of interest.

## Abbreviations

ALIS: aggresome-like induced structures
AP: alternative pathway
ATP: adenosine triphosphate
CNS: central nervous system
CO: carbon monoxide
DAMPs: damage-associated molecular patterns
EC: endothelial cell
ferrylHb: ferryl hemoglobin
FT: ferritin
FTH: ferritin heavy chain
GPXs: glutathione peroxidases
Hb: hemoglobin
HO: heme oxygenase
H_2_O_2_: hydrogen-peroxide
Hp: haptoglobin
Hsp: heat shock protein
Hx: hemopexin
H3K9ac: histone 3 acetylation at lysine-9
H3K27ac: histone 3 acetylation at lysine-27
ICAM-1: intracellular adhesion molecule-1
ICH: intracerebral hemorrhage
IgG: immunoglobulin G
IL: interleukin
LPS: lipopolysaccharide
MAC: membrane attack complex
metHb: met(ferric) hemoglobin
MyD88: myeloid differentiation primary response gene 88
NADPH: nicotinamide adenine dinucleotide phosphate
NET: neutrophil extracellular trap
NLR: NOD-like receptor
NLRP3: NLR family pyrin domain containing 3
NO: nitric oxide
NOD: nucleotide-binding oligomerization domain
NOX: NADPH oxidase
PAMPs: pathogen-associated molecular patterns
PMNs: polymorphonuclear cells
PPARγ: Peroxisome proliferator-activated receptor γ
PRR: pattern recognition receptor
PRXs: peroxiredoxins
RBC: red blood cell
ROS: reactive oxygen species
SAH: subarachnoid hemorrhage
SOD: superoxide dismutase
Syk: spleen tyrosine kinase
TLR: toll-like receptor
TNF-α: tumor necrosis factor-alpha
TRIF: TIR-domain-containing adapter-inducing interferon-β
VCAM-1: vascular cell adhesion molecule-1.
